# Urinary Tract Infection Due to Raoultella planticola in a Healthy Adult: A Report of a Rare Case

**DOI:** 10.7759/cureus.83046

**Published:** 2025-04-26

**Authors:** Emiliya Apinova, Robert C Bowers, Alex Somerville

**Affiliations:** 1 Obstetrics and Gynecology, West Virginia School of Osteopathic Medicine, Lewisburg, USA; 2 Obstetrics and Gynecology, Holzer Health System, Gallipolis, USA; 3 Family Medicine, Holzer Health System, Gallipolis, USA

**Keywords:** acute cystitis, emerging pathogen, klebsiella spp., raoultella planticola, urinary tract infection

## Abstract

A 56-year-old immunocompetent woman presented with recurrent urinary tract infections (UTIs) over a two-year period, during which *Raoultella planticola* was uniquely identified in her urine culture. Recurrent UTIs are generally defined as two or more infections within six months, or three or more within one year, and often prompt further investigation to identify potential risk factors. This case report highlights the rarity of *R. planticola* as a causative agent of UTIs and underscores the importance of comprehensive microbiological evaluation in patients with recurrent infections. The patient was successfully treated with trimethoprim-sulfamethoxazole, and the antimicrobial susceptibility profile of the isolate is discussed. This case contributes to the limited body of literature on *R. planticola*-associated infections and emphasizes the need for clinicians to remain vigilant for this emerging uropathogen, particularly in cases of recurrent cystitis.

## Introduction

*Raoultella planticola* is a gram-negative, nonmotile, aerobic rod belonging to the Enterobacteriaceae family [1]. It was originally classified within the genus *Klebsiella* and known as *Klebsiella planticola*; however, advancements in molecular techniques, particularly *16S rRNA* and *rpoB* gene sequencing, led to its reclassification under the genus *Raoultella* in 2001 [2,3]. *Raoultella planticola* is commonly isolated from environmental sources such as water, soil, and plants [1]. It is generally regarded as a nonpathogenic organism and is rarely implicated in human infections [1].

We report a rare case of a urinary tract infection (UTI) caused by *R. planticola* in a 56-year-old healthy woman. This case is notable due to the scarcity of documented instances linking *R. planticola* to lower urinary tract infections, particularly cystitis. While a few case reports have described *R. planticola* infections presenting with diverse clinical manifestations, its role as a uropathogen remains exceedingly rare. To the best of our knowledge, only three cases of *R. planticola*-associated UTIs have been reported in the United States to date.

## Case presentation

This case presents a 56-year-old postmenopausal woman who sought medical evaluation for recurrent urinary tract infections persisting over a two-year period. Her history was notable for previous UTIs caused by *Enterococcus faecalis* and *Klebsiella pneumoniae*. At presentation, she reported dysuria, left-sided abdominal pain, malodorous urine, hematuria, and mucus in the urine. On physical examination, the patient was afebrile with stable vital signs. Abdominal examination revealed mild left lower quadrant tenderness without rebound or guarding.

The patient's past medical history included diverticulosis, nephrolithiasis, and chronic constipation. Her surgical history was significant for rectocele and cystocele repair. Given that her current symptoms closely resembled her previous episodes of nephrolithiasis, further imaging was pursued. A computed tomography (CT) scan was performed at an outside facility to evaluate for renal calculi; however, no evidence of stones was found. Additionally, a prior cystoscopy had yielded normal results.

Urinalysis with culture was obtained to confirm a urinary tract infection. The urinalysis was notable for the presence of leukocyte esterase, nitrites, and moderate pyuria, consistent with a urinary tract infection. Routine blood work, including serum creatinine and a complete blood count, was within normal limits. The urine culture revealed *Raoultella planticola* at >100,000 colony-forming units per milliliter (CFU/mL). The antibiotic susceptibility profile of the isolate is presented in Table 1. The identification of this uncommon uropathogen emphasizes the importance of comprehensive microbiological evaluation in patients with recurrent UTIs. Based on the sensitivity results, the patient was treated with trimethoprim-sulfamethoxazole (Bactrim), 800 mg daily for seven days. Following the antibiotic course, the patient reported the complete resolution of symptoms with no recurrence at a one-month follow-up.

**Table 1:** Antibiotic Susceptibility Profile of Raoultella planticola

Antibiotic	Interpretation
Amikacin	Sensitive
Ampicillin	Resistant
Ampicillin/sulbactam	Sensitive
Cefazolin	Sensitive
Cefepime	Sensitive
Cefoxitin	Sensitive
Ceftazidime	Sensitive
Ceftriaxone	Sensitive
Ciprofloxacin	Sensitive
Gentamicin	Sensitive
Levofloxacin	Sensitive
Meropenem	Sensitive
Nitrofurantoin	Sensitive
Piperacillin/tazobactam	Sensitive
Trimethoprim/sulfamethoxazole	Sensitive

## Discussion

Urinary tract infections can range from asymptomatic bacteriuria to complicated ascending infections that may lead to bacteremia and sepsis. *Raoultella* spp. are phenotypically and molecularly similar to *Klebsiella* spp. [4]. While both genera are known to colonize humans and animals, they are infrequently associated with UTIs [5]. Studies suggest that between 9% and 18% of humans are colonized by *Raoultella planticola* [5], with the gastrointestinal and upper respiratory tracts serving as typical reservoirs [6].

Although infections caused by *R. planticola* are rare, cystitis appears to be the most commonly reported clinical manifestation [7]. Notably, type 1 fimbriae and mannose-sensitive hemagglutinins, virulence factors shared by *Klebsiella* and *Raoultella*,* *play a crucial role in the development of UTIs [8]. The similarity in these virulence mechanisms may provide insight into their overlapping pathogenic profiles.

The phenotypic and biochemical similarities between *Raoultella* and *Klebsiella* species make accurate differentiation difficult in routine clinical practice. Molecular techniques such as *16S rRNA* and *rpoB* gene sequencing are often required for definitive identification [9]. However, certain biochemical features can aid in distinction: *R. planticola* typically ferments lactose and is positive for citrate utilization and urease production, negative for oxidase, and capable of growth at 4°C [9]. Additionally, *R. planticola* can produce histamine via histidine decarboxylation, a property not shared with *Klebsiella* [10]. The awareness of these parameters can assist in accurate microbiological identification, especially when molecular tools are unavailable.

Advanced age, immunosuppressive conditions such as malignancy, diabetes mellitus, and renal impairment have been reported as risk factors for *R. planticola*-related UTIs [6,11]. These are consistent with risk factors for UTIs caused by other opportunistic pathogens [11]. However, our patient was an immunocompetent woman with no comorbid conditions, which makes this case particularly unusual. She had no known high-risk environmental exposures, such as recent travel, gardening, or contact with contaminated water sources, that could have served as likely reservoirs for *R. planticola*, further reducing the probability of environmental acquisition. The occurrence of recurrent urinary tract infection caused by *R. planticola* in a healthy host challenges the existing understanding of its pathogenic behavior and suggests that *R. planticola* may be capable of causing infections independently of host immunosuppression.

Although *R. planticola* has a broad tissue tropism, its pathogenesis is not yet well understood. Reported infections have included pneumonia, cholangitis, conjunctivitis, peritonitis, necrotizing fasciitis, cellulitis, bacteremia, and soft tissue infections [12].

Importantly, *R. planticola* exhibits intrinsic resistance to ampicillin due to the chromosomal expression of a class A β-lactamase [13]. Nonetheless, it generally remains susceptible to several antimicrobial classes, including aminoglycosides, carbapenems, cephalosporins, and fluoroquinolones [2]. This susceptibility profile makes these agents viable options for empirical and targeted therapy. However, the organism's ability to acquire plasmid-mediated resistance genes, particularly in hospitalized or immunocompromised patients, underscores the need for caution [14]. Given its evolving resistance potential and clinical unpredictability, *Raoultella planticola* should be considered in patients with recurrent UTIs, especially when standard pathogens are not isolated. Prompt identification and antibiotic stewardship are essential for effective treatment and to mitigate emerging antimicrobial resistance.

The incidence of *Raoultella planticola* urinary tract infections in the United States remains poorly defined, largely due to underreporting and frequent misidentification as *Klebsiella* species, given their close resemblance. However, only a small number of case reports, specifically three documented cases in the United States, have highlighted the ability of *Raoultella planticola* to cause clinically significant urinary tract infections. For instance, Skelton et al. reported a case involving a 73-year-old woman with multiple myelomas who developed a urinary tract infection caused by *R. planticola*, which was confirmed by urine culture and successfully managed with ceftriaxone [15]. Similarly, Howell and Fakhoury described a symptomatic UTI in a previously healthy two-month-old infant [8]. Richards and Musial documented a case of a mildly immunocompromised adult in whom *R. planticola* was isolated from urine culture and effectively treated with amoxicillin/clavulanate [16]. Our case contributes to the growing but limited literature by further establishing *Raoultella planticola* as a potential uropathogen in healthy adult patients. It highlights the importance of precise microbial identification and increased clinical awareness of this often-overlooked organism, particularly in cases of recurrent or atypical urinary tract infections.

## Conclusions

While *Raoultella planticola* is typically considered a rare, low-pathogenicity environmental organism, this case shows that it can cause meaningful infections even in healthy individuals. Though it is more often seen in immunocompromised patients, our findings suggest that it should not be overlooked in otherwise well patients with recurrent or unexplained urinary tract infections. As diagnostic methods improve and more cases come to light, clinicians should keep *R. planticola* in mind as a possible, though uncommon, uropathogen.
